# Nigral neuropathology of Parkinson’s motor subtypes coincide with circuitopathies: a scoping review

**DOI:** 10.1007/s00429-022-02531-9

**Published:** 2022-07-19

**Authors:** Jackson Tyler Boonstra, Hugo McGurran, Yasin Temel, Ali Jahanshahi

**Affiliations:** 1 School for Mental Health and Neuroscience, Maastricht University, Maastricht, Netherlands; 2 Department of Neurosurgery, School for Mental Health and Neuroscience, Maastricht University Medical Center, Peter Debyelaan 25A, 6229 HX Maastricht, The Netherlands; 3 Institute of Cell Biology and Neurobiology, Charité-Universitätsmedizin Berlin, Corporate Member of Freie Universität Berlin, Humboldt-Universität zu Berlin, Berlin Institute of Health, Berlin, Germany; 4 Charité-Universitätsmedizin Berlin, Corporate Member of Freie Universität Berlin and Humboldt-Universität zu Berlin, Einstein Center for Neurosciences Berlin, Charitéplatz 1, 10117 Berlin, Germany; 5 Netherlands Institute for Neuroscience, Royal Netherlands Academy of Arts and Sciences, Amsterdam, Netherlands

**Keywords:** Parkinson’s disease, Motor subtypes, Substantia nigra, Circuitopathies, Pathology, Scoping review

## Abstract

The neuropathological substrates of Parkinson’s disease (PD) patients with motor subtypes tremor-dominance (TD), non-tremor dominance (nTD), postural instability and gait difficulty (PIGD), and akinetic-rigid (AR) are not completely differentiated. While extensive pathological research has been conducted on neuronal tissue of PD patients, data have not been discussed in the context of mechanistic circuitry theories differentiating motor subtypes. It is, therefore, expected that a more specific and tailored management of PD symptoms can be accomplished by understanding symptom-specific neuropathological mechanisms with the detail histology can provide. This scoping review gives an overview of the literature comparing TD and nTD PD motor subtypes by clarify observed pathology with underlying physiological circuitry theories. Studies using an array of pathological examination techniques have shown significant differences between TD and nTD PD subtypes. nTD PD patients show higher neuronal loss, gliosis, extraneuronal melanin deposits, and neuroaxonal dystrophy in multiple subregions of the substantia nigra (SN) related to the overactivity of the indirect motor loop. TD patients show more severe cell loss specifically in medial SN subdivisions, and have damage in the retrorubral field A-8 that projects to the dorsolateral striatum and ventromedial thalamus in the direct motor loop. Pathological studies are consistent with neuroimaging data and support contemporary mechanistic circuitry theories of PD motor symptom genesis. Further multimodal neuroimaging and histological studies are required to validate and expand upon these findings.

**Supplementary Information:**

The online version contains supplementary material available at 10.1007/s00429-022-02531-9.

## Introduction

Parkinson’s disease (PD) is a progressive neurodegenerative disorder that affects more than 10 million people worldwide (Marras et al. 2018). Clinical diagnoses of PD are based on the identification of cardinal motor symptoms including bradykinesia, postural instability, rigidity, and tremor. Motor symptom subtypes can be clinically determined with the Movement Disorder Society Unified Parkinson’s Disease Rating Scale (MDS-UPDRS), an assessment of motor and non-motor PD symptoms (Goetz et al. 2007). The ratio of the mean tremor score to the mean score of their postural instability and gait difficulty (PIGD) score can be used to differentiate tremor-dominance (TD) patients (ratio ≥ 1.5), from PIGD patients (ratio ≤ 1), and from intermediate or ‘mixed-type’ patients (ratios > 1.0 and < 1.5) (Jankovic et al. 1990). Those patients diagnosed with PIGD have been called akinetic-rigid (AR) with both subtypes often denoted as non-tremor dominant (nTD).

Heterogeneous motor symptom presentation between PD motor subtypes suggest correspondingly diverse pathophysiologies (He et al. 2017; Rajput et al. 2009; van Rooden et al. 2010; Zaidel et al. 2009). Previous research reviews both TD and nTD PD patients differing in various neuroanatomical and neurofunctional domains (Boonstra et al. 2021) as well as in non-motor symptoms and quality of life (Wu et al. 2016). Patients with nTD have shown accelerated motor, functional, and cognitive decline compared to TD, resulting in TD to be considered the less severe subtype (Wu et al. 2016). In line with differing symptoms and disease severity, histological data show TD and nTD subtypes have ‘*specific morphological lesion patterns of pathophysiological relevance’* (Jellinger and Paulus 1992; Paulus and Jellinger 1991). It is, therefore, expected that a more specific and tailored management of PD symptoms can be accomplished by understanding symptom-specific neuropathological mechanisms with the detail histology can provide. Pathological examinations further contribute to the validation of in vivo neuroimaging readouts by virtue of resolution as the imaging modalities assist each other in a complementary nature.

Despite a plethora of research investigating the pathophysiological bases of PD (Borghammer 2018; Braak et al. 2003; Hornykiewicz 1998), particularly regarding the degeneration of midbrain dopamine (DA) neurons in the substantia nigra pars compacta (SNc), the full extent of PD pathology is poorly understood and even less is known about pathological differences between PD motor subtypes. Moreover, how previously described pathophysiological alterations between PD motor subtypes fit into contemporary mechanistic circuitry theories of PD symptoms genesis has yet to be described. The aim of this scoping review is to fill the gap between pathology and circuitry by consolidating previous studies that characterize pathological variances between TD and nTD subtypes of PD with contemporary mechanistic circuitry theories of PD symptom genesis.

## Methods

We conducted and reported this study adhering to the PRISMA-ScR (PRISMA Extension for Scoping Reviews) guidelines and following the scoping review framework developed by Arksey and O’Malley and advanced by Levac et al. (Arksey and O'Malley 2005; Levac et al. 2010), a six-stage methodological framework involving (1) identifying the research question, (2) searching for relevant studies, (3) selecting studies, (4) charting the data, (5) collating, summarizing, and reporting the results, and (6) consulting with stakeholders.

### Identifying relevant studies

A PubMed string search was iteratively developed with keywords “Parkinson's disease”, “tremor-dominant”, and “neuropathology” and variations thereof. The final string was used to identify all English-language abstracts containing human pathological data comparing the TD subtype to nTD subtype(s) within the NBIC database published up to December 2020. Additional articles were found from manual collation and supplemented references. The search string is provided in the Supplementary Material.

### Selecting studies and charting the data

Eligibility was assessed via abstract and full-text screenings. Articles containing human pathological data comparing the TD PD subtype to the nTD subtype(s) directly were taken for further review. Data from all eligible studies pertaining to the tremor dominant, non-tremor dominant, and healthy control sample sizes, methodology used during pathological data collection, and conclusionary results were extracted and tabled.

### Collating, summarizing, and reporting results

Results are grouped in the text by cardinal PD hallmarks including the dysfunction of the substantia nigra (SN) and dopaminergic system and the aggregation of Lewy bodies and amyloid-β plaques. We first discuss basal ganglia circuitry and the data, then describe how results coincide with neuroimaging research and fit into circuitopathy theories, as well as future clinical implications. While critical appraisal of studies is not required for scoping reviews, the main source of bias we identified were potential false positives stemming from small sample sizes. Additional plausible sources of bias include differential use of the MDS-UPDRS; practitioner- and patient-reported outcomes may vary between consolidated studies.

### Consulting with relevant stakeholders

It is recommended under the scoping review guidelines to involve stakeholders throughout the review process (Levac et al. 2010). During the design and implementation, our project team regularly discussed the review strategy and our interpretations with experts in various clinical, technical, and academic fields including with authors of the reviewed studies. While there were no patients involved with this review, results will be shared with any patients involved in future clinical exercises supported from this work.

### The substantia nigra

The SN is a bilateral nucleus in the basal ganglia (BG*)* and plays an important role in modulating movement. As the SN is a major source of dopamine, degeneration of dopamine neurons in the SN is the main pathological hallmark of PD (Alexander 2004). The SN can be divided into two subsections, the pars reticulata (SNr) that predominantly contains gamma-aminobutyric acid (GABA)-ergic neurons, mainly serving as an output nucleus in the cortico-basal ganglia loop conveying signals to the thalamus, superior colliculus, and to midbrain motor nuclei, and the pars compacta (SNc) projecting along the nigrostriatal pathway to the dorsal striatum (i.e., the caudate nucleus and putamen). SNc subdivisions innervate ventrally to the adjacent SNr (Haber and Knutson 2010) and modulate dopaminergic activity via axon collaterals. Fibers in the SNc have been shown to travel on average in a superior-posterior direction, whereas those in the SNr travel more in superior-medial/inferior-lateral directions (Plantinga et al. 2016). Medial, dorsal, and ventral SN dopaminergic regions generally project to the central and dorsal caudate, while caudal, lateral, and ventrolateral SN regions project more to the putamen (Bernheimer et al. 1973; Haber and Knutson 2010; Halliday et al. 1996; Jellinger 1999; Rinne et al. 1989).

The ventral region of the SNc is more vulnerable in numerous neurodegenerative diseases compared to the dorsal region partly due to the ventral tier being less melanised; vulnerability is concentrated ventrolaterally in PD and ventromedially in progressive supranuclear palsy (PSP) (Gibb and Lees 1991; Halliday et al. 1996). Functional neuroimaging has identified a tripartite connectivity-based parcellation of the SN; the *medial SN* functionally correlated to limbic striatal and cortical regions, the *ventral SN* to associative regions of cortex and striatum, and the *lateral SN* to somatomotor regions of striatum and cortex (Zhang et al. 2017). The medial SN could overlap with ventral tegmental area (VTA) as the VTA similarly projects via mesolimbic and mesocortical pathways to limbic and cortical areas. Due to a lack of clear tissue boundaries, this is sometimes referred to as the ‘SN/VTA’ complex.

### Parkinsonian circuitry models; direct, indirect, hyperdirect pathways

Motor circuitry organization of BG pathways include the *direct* pathway (striatum-SNr/globus pallidus interna (GPi)-thalamus-cortex, responsible for motor excitation), the *indirect* pathway (striatum-globus pallidus externa (GPe)-subthalamic nucleus (STN)-SNr/GPi-thalamus-cortex, for motor inhibition), the *hyperdirect* pathway (STN-SNr/GPi-thalamus-cortex, as baseline motor inhibition), and the nigrostriatal projection (SNc-striatum, that is modulatory) (Neumann et al. 2018). The striatum is the main input for the SNr via the *direct* (striatum-SNr) and *indirect* (striatum-GPe-STN-SNr) pathways. However, the *direct* and *indirect* pathways are neurochemically distinct; *direct* pathway neurons express high levels of DA subtype-1 receptor (D1) while neurons in the *indirect* pathway express more D2 receptors (Cazorla et al. 2015). Because of this, striatal activity via the *direct* pathways has an inhibitory effect on the SNr, while *indirect* pathway activity has an excitatory effect; a decrease in striatal DA leads to hyperactivity in D2 containing neurons and hypoactivity in D1 neurons (Cazorla et al. 2015). Therefore, it was generally thought that hypotonic-hyperkinetic symptoms of PD such as tremors are caused by dysfunction of the *indirect* loop, leading to the loss of movement inhibition, while hypertonic-hypokinetic symptoms such as bradykinesia (a slowing of movement) are caused by dysfunction of the *direct* pathway that typically serves to plan and execute movements (Obeso et al. 2000), but neuropathological evidence for this is inconclusive.

Two classical functional hypotheses explaining the pathophysiological circuitry underlying movement dysfunction in PD include the ‘firing rate’ model and the ‘firing pattern’ model (Nambu et al. 2015). In the ‘firing rate’ model, dopamine depletion in the BG reduces excitation to striatal *direct* pathways that project to the GPi and reduces tonic inhibition to striatal *indirect* pathways that project to the GPe. This leads to an increase in the firing rates of the GPi and SNr via the inhibition of the striato-GPi/SNr *direct* pathway, excitation of the striato-GPe-STN-GPi/SNr *indirect* pathway, and subsequent decreased thalamic and cortical activity resulting in akinesia. A key limitation of the ‘firing rate’ model is that it does not explain tremor circuitries. The ‘firing pattern’ model suggests oscillatory and/or synchronized firing patterns in the BG disables the processing and relaying of motor-related information leading to movement dysfunction. These abnormal firing patterns exist in the form of bursts (a series of firings in short time periods) and oscillations (periodic bursts, ranging from 4 to 9 Hz ‘tremor frequency’ and 10 to 30 Hz ‘beta frequency’) (Ashkan et al. 2017). It is thought that dopamine depletion could enhance reciprocal connections between the GPe and the STN and lead to increased synchronized oscillatory activity resulting in motor dysfunction (Hutchison et al. 2004). While the ‘firing pattern’ model partially explains tremors, its explanation for akinesia is less clear.

**Fig. 1:**
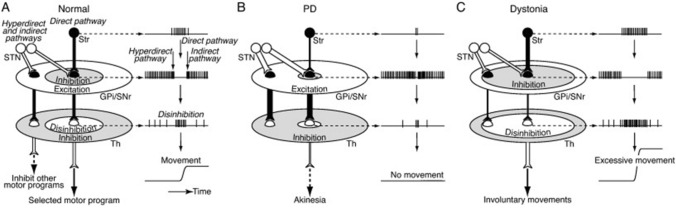
*‘Dynamic activity model’* diagraming voluntary control of movement. ** A** Normal state, **B** Parkinson's disease, **C** Dystonia. Spatial distributions (left) and temporal patterns (right) of neuronal activity are illustrated in each panel. STN; subthalamic nucleus, GPi/SNr; internal global pallidus/substantia nigra, Th; thalamus. Reprinted with permission from Nambu et al. (2015)

Nambu et al. (2015) proposed a novel *‘dynamic activity’* model to reconcile the *firing rate* and *firing pattern* models, where signals from the *direct* pathway disinhibit thalamic neurons in a *center* thalamic area, *hyperdirect* and *indirect* pathways inhibit the *center* area, and *hyperdirect* and *direct* pathways inhibit the *surround* thalamic area (Fig. 1) (Nambu et al. 2015). The *center-surround* composition is based on an anatomical study showing excitatory STN–GPi fibers arborize more extensively and terminate more proximally in the thalamus than inhibitory striato–GPi fibers (Parent and Hazrati 1995), although the authors acknowledge that different studies suggest highly specific STN–GPi and striato-GPi projections (Smith et al. 1998; Shink et al. 1996). In this model, dopamine depletion is thought to reduce GPi inhibition via the *direct* pathway to the *center* area and facilitate GPi excitation via the *hyperdirect* and *indirect* pathways to the *center* and *surround* areas, leading to a reduction of thalamic and cortical disinhibition and subsequent akinesia. Furthermore, hyperkinetic disorders like tremors are said to result from the combination of enhanced inhibition to the *center* area of the GPi via the *direct* pathway and reduced excitation via the *hyperdirect* and *indirect* pathways to the *center* and *surround* area causing uncontrolled thalamic and cortical disinhibition. Further electrophysiological and anatomical studies investigating movement-related BG activity are needed to verify the *‘dynamic activity’* model. Moreover, how specific pathological differences between PD motor subtypes correspond to circuitry theories are prominently missing from all models.

## Results

### Basal ganglia degeneration between subtypes

An array of quantitative histological differences in basal nuclei degeneration have been shown between PD motor subtypes (Table 1). Histological research examining extensive clinical documentation of 45 autopsy confirmed PD patients with AR subtype show higher neuronal loss in the medial and lateral SN (devised by a line through the cerebral aqueduct) and the locus coeruleus (LC) compared to TD, as well as more severe SN gliosis, extraneuronal melanin deposits, and neuroaxonal dystrophy (Paulus and Jellinger 1991). In this study, neuroaxonal dystrophy of the SNr was seen in 10 out of 28 AR patients but in none of the 15 TD patients, while neuronal reduction in the dorsal raphe nucleus (DRN) was equal between subtypes (Paulus and Jellinger 1991).

**Table 1:** Neuropathological variances in Parkinson’s disease motor subtypes

Article	Patient numbers per subtype							Methods	Findings
	PD	TD	nTD	PIGD	AR	Mixed	HC		
Bernheimer et al. (1973)	28						28	IHC	Degree of akinesia correlated with DA and HVA decreases in the caudate nucleus, while the degree of tremor matched best with the degree of HVA decrease in the pallidum
Rinne et al. (1989)	12						18	IHC	Positive association found between rigidity and hypokinesia and low neuronal densities in the SNL
Paulus et al. (1991)		18			27			IHC	AR had a higher degree of gliosis in the SNM and SNL and more intense extraneuronal melanin deposits in the SNL. Neuroaxonal dystrophy of the SN reticula was seen in 10/28 AR but not in any of the 15 TD. AR have higher neuronal loss in the SNL and SNM and LC. Neuronal reduction in the DRN did not differ between AR and TD
Jellinger and Paulus (1992)		18			27			IHC	AR show degeneration of the striatonigral dopaminergic system and neuronal loss in SNL, SNM, and noradrencrgic LC. Tremor was related to other anatomo-pathophysiological substrates
Rajput et al. (1993)		34		11				x	The majority of tremor-onset cases had Lewy body disease while the majority of PIGD-onset cases had other forms of pathology
Halliday et al. (1996)					20		15	IHC	Greater dorsomedial SN cell loss was seen in PD patients with resting tremor than in those without tremor (85% loss compared to 70%, respectively)
McRitchie et al. (1997)					7		5	IHC	A8 regions appear largely unaffected in AR PD
Rajput et al. (2008)		3			3	2	5	HPLC	AR have greater loss of DA in both the dorsal and ventral regions of the rostral GPi compared to TD and Mixed
Selikhova et al. (2009)	242	88	93					Review	nTD have higher mean pathological grading of cortical Lewy bodies, more cortical amyloid-b plaque load, and more cerebral amyloid angiopathy compared to the EDO onset group and to TD. nTD have higher burdens of cortical Lewy bodies in the frontal regions and more severe plague formation in the neocortex compared to EOD and TD. EOD and TD were more likely to have brainstem and limbic Lewy body disease compared to nTD. Neocortical Lewy body class was more related to sever bradykinesia and falls than localized or limbic Lewy body disease was
Selikhova et al. (2013)	15	15 BT PD						IHC	Benign tremulous PD had less global neuronal loss in the substantia nigra compared to pathological controls. Benign tremulous PD had less cell loss in all nigral subregions compared to pathological controls. The most severe cell loss was seen in the ventrolateral nigra, while the medial nigra showed the greatest difference between benign tremulous PD and pathological controls
De Pablo-Fernandez et al. (2019)	111							Review	Staging of Lewy pathology and Alzheimer disease–related pathology did not differ between unique PD subtypes, but showed different rates of progression

*A8* dopaminergic cell group A8, *AR* akinetic-rigid, *BT* benign tremulous, *DA* dopamine, *DRN* dorsal raphe nucleus, *EOD* early onset disease, *GPi* global pallidus internal, *HC* healthy control, *HPLC* high-performance liquid chromatography, *HVA* homovanillic acid, *IHC* immunohistochemistry, *LC* locus coeruleus, *Mixed* mixed-ratio subtype, *nTD* non-tremor dominant, *PD* Parkinson’s disease, *PIGD* postural instability and gait difficulty, *SNL* substantia nigra lateral, *SN* substantia nigra, *SNM* substantia nigra medial, *TD* tremor-dominant

In another study on pathologically confirmed PD patients with severe Parkinsonian AR syndrome, examinations showed that cell loss in the dorsolateral SN correlated with the duration of PD symptoms, while cell loss in the dorsomedial SN correlated with the presence of tremor (Halliday et al. 1996). TD showed more neuronal loss in the medial rather than in the lateral SNc, an area that projects to the caudate nucleus and anterior putamen (Jellinger 1999). The ventrolateral part of the SNc typically degenerates faster in AR PD relative to the medial SNc, with negative correlations between SNc cell counts, severity of AR symptoms, and dopamine loss in the posterior putamen (Jellinger 1999). When quantifying neuronal densities from the medial to lateral part of the SN in idiopathic PD brains and controls, a positive association was seen between rigidity and hypokinesia and low neuronal densities in the lateral SN, but tremor was less severe in patients with similarly low neuronal densities (Rinne et al. 1989). As the SN is the neuronal region where the enzyme tyrosine hydroxylase (TH) is most abundant, TH deficiency has shown to be associated with hypokinetic-rigid symptoms while other features like tremor are largely absent in patients with TH deficiency (Willemsen et al. 2010).

Although TD patients have shown less severe total SN cell loss (60%) compared to nTD (68%), TD patients have more cell loss in the periretrorubal field A8 in the lateral reticular formation (Hirsch 1991). A8 is located in the midbrain reticular formation dorso-caudal laterally to the SN and projects to the dorsolateral striatum and ventromedial thalamus (Deutch et al. 1988). Interestingly, in a study of seven autopsy confirmed AR patients with resting tremor, the A8 region appeared to be largely unaffected, with equal volume and neuronal count between the PD cases (those with only nigrostriatal pathway degeneration), Parkinson plus syndromes (those with additional degeneration elsewhere), or healthy controls (McRitchie et al. 1997). The degree of PD akinesia has been shown to correlate with decreased dopamine and its inactive metabolite homovanillic acid in the caudate nucleus, while tremor severity correlates more with reduced homovanillic acid in the pallidum (Bernheimer et al. 1973). Additionally, using high-performance liquid chromatography, AR patients have shown to have a greater loss of dopamine in both the dorsal and ventral regions of the rostral GPi compared to TD and mixed subtypes (Rajput et al. 2008).

It is clear that quantitative differences in BG degeneration are visible on histological levels between PD motor subtypes. Pathological results indicate a multitude of nigrostriatal degenerative processes innate to PD are less severe in TD compared to nTD; nTD patients show more severe degeneration in the lateral parts of the SN, while TD patients show more alterations in medial SN regions.

### Cortical Lewy bodies and amyloid-β plaques between subtypes

Parkinsonian diseases involve abnormal accumulation of α-synuclein proteins within neuronal, glial, and nerve fiber cells. The progressive aggregation of Lewy bodies (LB), Lewy neurites, and amyloid-β plaques (Aβ) are additional hallmark histological markers of PD (Alexander 2004). In 1 study of 70 autopsy-verified PD patients, the majority of tremor-onset cases (55.9%) had LB disease while the majority of PIGD-onset cases (73%) had other or additional forms of pathology including multiple system atrophy and PSP (Rajput et al. 1993). This study aimed to evaluate the mode of onset and prognosis in autopsy confirmed PD cases and showed PIGD manifestations in PD were not due to age of onset as previously thought, but resulted from dissimilar neuropathology.

nTD patients have shown higher mean pathological grading of cortical LB, more cortical Aβ load, and more cerebral amyloid angiopathy compared to early-onset disease (EOD, < 45 years of age) and TD patients (Selikhova et al. 2009). In this study, the age of death was equal between cohorts indicating the higher cortical LB seen in nTD were not effects of age. Additionally, nTD showed higher cortical LB in the frontal regions and more severe plaque formation in the neocortex compared to EOD and TD who were more likely to have brainstem and limbic LB disease. Neocortical LB class was also related more to severe bradykinesia and falls than localized or limbic LB disease was (Selikhova et al. 2009). In De Pablo-Fernández et al., the staging of Lewy pathology and Alzheimer’s disease-related pathology did not differ between PD subtypes, but showed different rates of progression (De Pablo-Fernandez et al. 2019). Although this study did not group according to TD and nTD subtypes, instead using mild-motor predominant, intermediate, and diffuse malignant groupings, 79% of those in the mild motor-predominant subtype were TD and 33% of the diffuse malignant subtype were PIGD, and it demonstrated that the speed of pathological accumulation in certain neuronal areas varies between PD subtypes (Fereshtehnejad et al. 2017).

From these studies, it appears that patients with the nTD PD subtypes suffer more from cortical LB depositions and carry additional diverse forms of pathologies while TD patients have more localized pathology in the brainstem and limbic LB disease. Using cluster analyses, the authors suspect that a spectrum exists between a SN origin and a multi-pathway diffuse neurodegenerative process that is coupled with individual variation in progression and pathological patterns (Fereshtehnejad et al. 2017). As PD pathology gradually progresses from the brainstem to higher cortical layers as the disease advances, nTD patients with comparable disease duration to TD patients may be demonstrating greater and faster degeneration along this specific network that is not simply due to age or different stages of the disease.

### Neuroimaging findings support pathology

Neuroimaging studies examining nTD and TD patients support findings of pathology variance in the SN. In Table 2, an overview is provided. Diffusion tensor imaging (DTI) measures water molecule diffusion and directionality and is an indirect marker for white matter composition. PD patients show reduced diffusion measurements in the ventrolateral SN in keeping with dopaminergic cell loss (Vaillancourt et al. 2009). Reductions of the DTI measurement fractional anisotropy (FA, reflecting a combination of axonal density, fiber mixture, and density) in the SN and putamen correlate with increased UPDRS motor scores in PD patients (Zhan et al. 2012). DTI has also shown increased mean diffusivity and radial diffusivity relating to structural disintegration in the SN being driven largely by the PIGD subtype (Nagae et al. 2016). Additionally, using neuromelanin-sensitive magnetic resonance imaging (NM-MRI) to detect dopamine metabolism, researchers found NM contrast-to-noise ratio (CNR) values in the lateral SN to decrease linearly with PD progression, while PIGD patients show larger decreases in CNR values in the lateral SN compared to TD (Wang et al. 2021). PIGD have also shown more severe NM signal attenuation in the medial SN compared to TD with the medial SN ipsilateral to the most clinically affected side showing the greatest power to discriminate PD motor subtypes (Xiang et al. 2017).

**Table 2:** Substantia nigra neuroimaging findings in Parkinson’s disease motor subtypes

Article	Patient numbers per subtype							Methods	Findings
	PD	TD	nTD	PIGD	AR	Mixed	HC		
[44]	14						14	DTI	Reduced FA in PD patients SN was greater in the caudal region compared with the middle and rostral regions
[45]	12						20	DTI	Alterations of FA in the SN and putamen correlate with increased UPDRS motor scores
[46]	9	12					20	DTI	Reduced FA and increased MD and RD in the SN was largely driven by PIGD. Increased diffusivity in the globus pallidus correlated with disease stage and motor severity in PIGD
[48]		9		14			20	NM-MRI	PIGD have more severe signal attenuation in the medial SN of compared to TD, with the medial SN ipsilateral to the MAH having the greatest power to discriminate PD motor subtypes
[49]	70						20	MR	SN MR phase shifts are positively correlated with UPDRS-III and bradykinesia-rigidity subscores but not with tremor subscores
[50]		10			10		20	MR	Positive correlation between motor subtype ratio and R2* in the putamen, caudate, and thalamus but not in SN, with larger TD ratios having higher R2*

*DTI* diffusion tensor imaging, *HC* healthy control, *Mixed* mixed-ratio subtype, *nTD* non-tremor dominant, *PD* Parkinson’s disease, *PIGD* postural instability and gait difficulty, *SN* substantia nigra, *TD* tremor-dominant, *MD* mean diffusivity, *RD* radial diffusivity, *MAH* most affected hemisphere, *MR* magnetic resonance imaging, *NM-MRI* neuromelanin-sensitive magnetic resonance imaging, R2* relaxometry rate, *FA* fractional anisotropy, *UPDRS* Unified Parkinson’s Disease Rating Scale

High-pass filtered phase MRI used to measure iron depositions show SN phase shifts to be positively correlated with UPDRS-III and bradykinesia-rigidity subscores but not with tremor subscores (Martin-Bastida et al. 2017). Additionally, MRI has found positive correlations between motor subtype ratio and measures of iron via transverse relaxation rates (R2*) in the putamen, caudate, and thalamus but not in the SN with larger TD motor phenotype ratios having higher R2* suggesting putaminal over nigral iron accumulation as an early TD predictor (Bunzeck et al. 2013). Taken together, these results suggest that regional patterns of microstructural degradation found via various neuroimaging techniques in the SN prove to accurately distinguish between PD motor subtypes and support histological findings assessing PD-related neurodegeneration. In short, neuroimaging shows nTD subtypes to have more laterally concentrated SN related severities compared to TD. Production of an accurate and detailed description of complete SN anatomy based on histological and immunohistochemistry stainings (e.g., Perl, Luxol fast blue/Cresyl violet, substance P, and Calbindin) of PD tissue conjointly imaged with high-field MRI (i.e., *MR microscopy*) has proven successful (Massey et al. 2017) warranting similar examination across PD subtypes.

How cortical neuritic plaque differences between PD subtypes correspond to neuroimaging data is less clear. Although MRI typically shows more severe cortical volumetric changes in PIGD compared to TD, on a whole volumetric variations between PD motor subtypes are mixed (Boonstra et al. 2021); TD patients have shown to have higher gray matter volumes (GMV) (Al-Bachari et al. 2017; Benninger et al. 2009; Herb et al. 2016; Piccinin et al. 2017), lower GMV (Rosenberg-Katz et al. 2013, 2016), and similar GMV (Karunanayaka et al. 2016; Linder et al. 2009; Nyberg et al. 2015; Prodoehl et al. 2013; Tessa et al. 2008; Vervoort et al. 2016) in cerebellular and cortical regions when compared to nTD subtypes. Correspondingly, greater attention should be granted towards cerebellular and brainstem pathways, as heterogeneous imaging acquisitions applied to all PD patients may lead to critical subcortical variations being overlooked. Parkinsonian resting tremor has shown to be related to cerebellar receiving thalamus activity (Helmich et al. 2012), although to our knowledge, no histological work has been done to support various neuroimaging studies that investigated cerebellar circuitopathies. This exact line of research will be a focus in our departments’ future work.

### Pathology supports circuitry models

Evidence from pathological research does not definitively support the notion that tremor symptoms of PD are mainly caused by dysfunction of the *indirect* loop while bradykinesia is caused by dysfunction of the *direct* pathway. nTD patients show more severe cell loss in the ventrolateral part of the SN that projects to the dorsal putamen, while SNc cell loss has also shown to negatively correlate to severity of akinesia-rigidity and DA loss in the posterior putamen (Jellinger 1999). The ventrolateral SNc in nTD has additionally shown to degenerate more severely than the medial SN (Bernheimer et al. 1973) in contrast to TD patients, who exhibit less severe total and lateral SNc cell loss, but more severe cell loss in the medial SNc, as well as damage to the A8 region not seen in AR/nTD (Damier et al. 1999; Deutch et al. 1988; Jellinger and Paulus 1992).

nTD SN deficits could underpin pathological hyperactivity of the GABAergic *indirect* motor loop. GABAergic projections of the putamen have an inhibitory effect on the thalamus providing neuronal deficits in SN areas that project to the putamen to cause pathological thalamocortical pathway inhibition leading to reduced cortical activation. TD patients show pathological deficits in matrices of the dorsolateral striatum and ventromedial thalamus *direct* motor loop that could relate to hyperactivity in thalamomotor and cerebellar projections underpinning tremors. Such differential degeneration seen between PD motor subtypes could prove to be anatomical support for the Nambu et al., 2015*‘dynamic activity’* model in how the GPi/SNr receives differential and competitive inputs from the *hyperdirect*, *direct*, and *indirect* pathways that modulate movement (Nambu et al. 2015). Variations within the proposed *center-surround* topographical distribution of striatum-derived inhibition on the GPi/SNr (Nambu et al. 2015) and its functional consequences could partly be explained by dissimilar neuropathological depositions in subpopulations of dopamine cells in the SN. nTD deficits in the ventrolateral SN could partly explain the proposed reduction in GPi/SNr inhibition in the *center* area and facilitation of GPi/SNr excitation in the *center* and *surround* areas that are suspected to lead to akinesia. Likewise, TD deficits in the medial SN could enhance inhibition in the *center* area of the GPi/SNr and reduced GPi/SNr excitation in the *center* and *surrounding* areas said to lead to involuntary movements.

Further research is needed to verify topographical distributions of SN subregion pathology. Investigations involving both subcortical and cortical stimulation are further required to functionally map striatum-derived inhibition and STN-derived excitation in the GPi to characterize ‘*dynamic activity’* alterations between PD motor subtypes (Nambu et al. 2015). Tracing studies in non-human primates using cell-type specific viral tools (El-Shamayleh et al. 2016) can also shed light on projection patterns of medial and lateral SN dopamine cells. Additionally, how basal–brainstem pathways relate to diverse symptoms of PD remains to be studied in equal detail.

### Clinical application

Modeling discrepancies between PD subtypes should have the ultimate aim to improve fundamental understandings of the disease and promote clinical success. Cortical pathological alternations could relate to the non-motor symptoms often seen more in nTD than in TD (Wu et al. 2016). In cognitively normal elderly individuals increased global amyloid pathology showed to be associated with alterations in gray matter networks indicative of network breakdowns that lead to dementia (Ten Kate et al. 2018). Although correlations of Aβ deposition with cognitive decline in PD is still a matter of debate (Compta et al. 2014; Shah et al. 2016; Melzer et al. 2019), a better understanding of variations in dopaminergic cell loss, plaque, and Lewy body depositions within PD patients has the potential to lead to more tailored treatment regimens and opens the opportunity to repurpose existing drug treatments, including dopamine (L-DOPA) based and amyloid targeted therapies (apomorphine) (Titova and Chaudhuri 2017).

Results hold further clinical implications in the field of deep brain stimulation (DBS) as TD patients tend to respond better to DBS, whereas those with axial subtypes and gait problems benefit less favorably (Neudorfer et al. 2019; Neumann et al. 2018; Xu et al. 2018) demonstrating a clear need for improved diagnoses between PD subtypes. The SN is being studied as a DBS target region for PD motor symptom alleviation (Sutton et al. 2013; Valldeoriola et al. 2019). Low-frequency SNr-DBS has shown to result in a significant improvement in PD patient’s freezing of gait, although its global antiparkinsonian effects were lower than that of the more common high frequency STN-DBS (Valldeoriola et al. 2019). STN-DBS has shown to have a protective effect on SNc neuron apoptosis in PD animal models conceivably resulting from neurotransmitter distribution and metabolism alterations (Wu et al. 2012), while supportive clinical data remain sparse.

### Clinical limitation

It should be noted that the PD motor subtypes of TD and nTD have shown to have substantial clinical variability over time, specifically for the PIGD subtype (Simuni et al. 2016). After a 4-year follow-up in 325 PD patients, the proportion of recorded PIGD patients was shown to have increased significantly, from 23.1% at baseline to 44.2%. (Lee et al. 2019). Controversies exist that TD and PIGD are not different PD subtypes but instead are different clinical PD stages with dissimilar disease progression features (Lee et al. 2019). Correspondingly, the positive correlation between SN neurons and tremor may have been due to patients having not yet shifted from a TD subtype to nTD (Rinne et al. 1989). It is then reasonable to question if conventional PD motor subtyping has proficient clinical applicability. While authors found that 79% of those in the mild motor-predominant subtype were TD and 33% of the diffuse malignant subtype were PIGD, the tremor/PIGD classifications alone could not predict prognosis unlike their global subtyping solution that integrated additional daily living and cognition factors (Fereshtehnejad et al. 2017). These results speak towards the utility of incorporating various motor and non-motor symptoms as well as other biomarkers in the identification of PD subtypes, as more multivariate groupings may produce greater clinical applications and prove more stable over time (Ren et al. 2021). It is still unknown why certain subpopulations of cells undergo different degeneration patterns within each PD subtype, what initiates these divergences, whether they are a cause or a consequence of other co-occurring pathogenic mechanisms, and how they may be best targeted for treatment.

## Conclusion

The purpose of scoping reviews is to chart a field within research and aid practitioners in constructing future research agendas (Arksey and O'Malley 2005). Clustering pathological differences between PD motor subtypes by combining MRI and histology to understand neuroanatomical pathways underlying clinical symptoms is essential in clarifying PD etiology. Literature displays a vast array of SN variation across PD subtypes, showing nTD to have more severe damage in the ventrolateral part of the SN while TD patients show less severe total SN cell loss but more severe medial SN alterations (Damier et al. 1999; Deutch et al. 1988; Jellinger and Paulus 1992). Patients with the nTD subtype further suffer from more cortical LB depositions and additional pathologies while TD patients have more brainstem and limbic LB disease (Rajput et al. 1993; Selikhova et al. 2009).

Neuroimaging studies support pathological findings between PD subtypes showing alterations within SN subsections (Martin-Bastida et al. 2017; Wang et al. 2021; Xiang et al. 2017), although support for cortical plaque depositions is less clear (Boonstra et al. 2021). Pathological differences between PD subtypes in the SN support the ‘*dynamic activity’* model of PD symptom genesis; SN pathology may sustain the proposed *center-surround* topographical distribution of striatum-derived inhibition on the GPi/SNr that is correlated to motor disabilities (Nambu et al. 2015). Findings hold clinical implications in support of pathocircuitries and towards novel targets for treatment and interventions (Sutton et al. 2013; Valldeoriola et al. 2019; Wu et al. 2012).

Translational neuroscience works to disentangle the relationship between pathological data found ex vivo and clinically relevant in vivo data to innovate towards earlier PD diagnostic tools, better monitoring, and improved treatment approaches. Further research should continue to improve upon the creation of sustainable multivariate PD subtypes. As histology confers specificity by virtue of resolution neuroimaging should be inspired by postmortem findings and direct examinations with greater detail towards distinct neuronal pathway deficits.

## Supplementary Information

Below is the link to the electronic supplementary material.

Supplementary file1 (DOCX 12 kb)

Supplementary file2 (TXT 130 kb)

## Data Availability

The datasets generated during and/or analysed during the current study are available in the text and Supplementary Materials.
