# Supplementary material for: Nigral neuropathology of Parkinson’s motor subtypes coincide with circuitopathies: a scoping review
Source: Brain Struct Funct. 2022 Jul 19;227(7):2231–42. doi: 10.1007/s00429-022-02531-9 (PMC9418085; doi:10.1007/s00429-022-02531-9)
Supplement: Supplementary file 1 — Supplementary file1 (DOCX 12 kb) [file 429_2022_2531_MOESM1_ESM.docx]

("parkinson disease"[MeSH] OR "parkinsonian disorders"[MeSH] OR "lewy body disease"[MeSH] OR ("parkinson disease"[MeSH terms] OR ("parkinson"[all fields] AND "disease"[all fields]) OR "parkinson"[All Fields] OR "parkinson disease"[all fields] OR ("parkinson's"[all fields] AND "disease"[all fields]) OR "parkinson's"[All fields])) AND (“pathology’[MeSH Terms] OR (“neuropathology”[MeSH Terms]) OR “neuropathology”[All Fields] OR (“neuropathologic”[All Fields] OR “neuropathological”[All Fields] OR “pathophysiology”[All Fields] OR “pathophysiological”[All Fields] OR “clinico-pathological”[All Fields]) OR (“histology”[All Fields] OR “histological”[All Fields] OR “histopathological”[All Fields] OR “biomarkers”[All Fields])) AND ("motor subtypes"[All Fields] OR ("motor"[All Fields] AND "subtype"[All Fields] OR "subtypes"[All Fields] OR "subgroups"[All Fields] OR "phenotype"[All Fields] OR "phenotypes"[All Fields]) OR ("postural instability and gait disorder"[All Fields] OR ("postural"[All Fields] AND "instability"[All Fields] AND "gait"[All Fields]) OR "PIGD"[All Fields]) OR ("akinetic-rigid"[All Fields] OR "akinetic"[All Fields] OR "rigid"[All Fields] OR "rigidity"[All Fields] OR "akinetic-rigidity"[All Fields] OR hypokinesia-rigidity[All Fields] AND ("tremor"[MeSH Terms] OR "tremor"[All Fields])) AND (("tremor"[MeSH Terms] OR "tremor"[All Fields]) AND dominant[All Fields]) AND tremor-dominant[All Fields] AND (non[All Fields] AND ("tremor"[MeSH Terms] OR "tremor"[All Fields]) AND dominant[All Fields]) AND non-tremor[All Fields]) AND ("humans"[MeSH Terms] AND English[lang])
